# Prof. Chapman on Medical Reform

**Published:** 1847-06

**Authors:** 


*Prof. Chapman on Medical Reform.*—The venerable and illustrious professor of the Practice of Medicine in the University of Pennsylvania, in the address delivered by him to the graduates of that school, touched in a very felicitous manner on the existing condition of the profession.

"By an extraordinary multiplication of medical schools," he remarked, "a vulgar rivalry has arisen in spirit and conduct similar to that displayed in the competitions of steamboats, and railroads, with the same means resorted to for reaching success. Education is cheapened, the period of study abridged, or lightened—no irksome examinations are to be endured, and degrees acquired easily and assuredly. The annual number of graduates, in medicine, is at present probably larger in the United States, than in the whole of the residue of the civilized world. Does not this startling fact sufficiently proclaim, that the most precious of professional honors, designed for the meritorious alone, is dispensed with criminal liberality? Confessedly the diploma has lost its value. Every one knows of its prostitution, and has ceased to regard it as in itself deserving of attention. What other conclusion could indeed be formed, when it cannot be denied that nearly every audacious empiric of the land, is sheltered under its protection, and enjoys its immunities and privileges?

"Effects alarmingly menacing to the elevation, and real usefulness, of our science, may be discerned, as proceeding from these abuses and corruptions. Degraded in its position and authority, by a forfeiture of the public confidence, it has become mean, abject, obsequious—courting favor by artifice, or means equally ignoble and detestable. Charlatanism has spread its infection through it, and with its hideous features it is impressed. Do I calumniate, exaggerate, or even paint too warmly? The indignant tones of every medical man, honest and intelligent, are only repeated. Nay, the glad tidings are abroad that, the profession no longer enduring its grievances, is determined to be heard, and with a potential voice. Three hundred delegates from its embodied masses, are at this moment speeding their way to this city, to meet in convention, to devise measures to eradicate these very evils, and redress other wrongs. Cordial will be our welcome to these missionaries of good, and hearty our co-operation with them, in every effort tending to the purification of medicine, or to its higher and more perfect glory."—*Medical News.*

